# A Mutational Analysis of the Endophilin-A N-BAR Domain Performed in Living Flies

**DOI:** 10.1371/journal.pone.0009492

**Published:** 2010-03-03

**Authors:** Anita G. Jung, Christina Labarerra, Anna M. Jansen, Klaus Qvortrup, Klemens Wild, Ole Kjaerulff

**Affiliations:** 1 Department of Neuroscience and Pharmacology, University of Copenhagen, Copenhagen, Denmark; 2 Department of Biomedical Sciences, University of Copenhagen, Copenhagen, Denmark; 3 Heidelberg University Biochemistry Center, Heidelberg, Germany; University of Birmingham, United Kingdom

## Abstract

**Background:**

Endophilin is a cytoplasmic protein with an important function in clathrin-dependent endocytosis at synapses and elsewhere. Endophilin has a BAR (Bin/Amphiphysin/Rvs-homology) domain, which is implicated in the sensing and induction of membrane curvature. Previous structure-function studies of the endophilin-A BAR domain have almost exclusively been made in reduced systems, either in vitro or ex vivo in cultured cells. To extend and complement this work, we have analyzed the role played by the structural features of the endophilin-A BAR domain in *Drosophila* in vivo.

**Methodology/Principal Findings:**

The study is based on genetic rescue of *endophilin-A* (*endoA*) null mutants with wild type or mutated *endoA* transgenes. We evaluated the viability of the rescuants, the locomotor behavior in adult flies and the neurotransmission at the larval neuromuscular junction. Whereas mutating the endophilin BAR domain clearly affected adult flies, larval endophilin function was surprisingly resistant to mutagenesis. Previous reports have stressed the importance of a central appendage on the convex BAR surface, which forms a hydrophobic ridge able to directly insert into the lipid bilayer. We found that the charge-negative substitution *A66D*, which targets the hydrophobic ridge and was reported to completely disrupt the ability of endophilin-BAR to tubulate liposomes in vitro, rescued viability and neurotransmission with the same efficiency as wild type *endoA* transgenes, even in adults. A similar discrepancy was found for the hydrophilic substitutions *A63S/A66S* and *A63S/A66S/M70Q*. The *A66W* mutation, which introduces a bulky hydrophobic side chain and induces massive vesiculation of liposomes in vitro, strongly impeded eye development, even in presence of the endogenous *endoA* gene. Substantial residual function was observed in larvae rescued with the *EndoA(Arf)* transgene, which encodes a form of endophilin-A that completely lacks the central appendage. Whereas a mutation (*D151P*) designed to increase the BAR curvature was functional, another mutation (*P143A*, *ΔLEN*) designed to decrease the curvature was not.

**Conclusions/Significance:**

Our results provide novel insight into the structure/function relationship of the endophilin-A BAR domain in vivo, especially with relation to synaptic function.

## Introduction

Endophilin is a highly conserved cytoplasmic protein involved in endocytotic membrane trafficking. The mammalian endophilin family includes endophilin-A 1–3 and endophilin-B 1–2 [reviewed in 1]. Endophilin-A is enriched at synapses and implicated in clathrin-dependent endocytosis, a major vesicle retrieval pathway active during and following exocytotic activity [2], [3], [4], [5], [6]. Endophilin-A has a C-terminal SH3 domain, which binds proline-rich domains of dynamin and synaptojanin, two major players in clathrin-dependent endocytosis [7]. Endophilin-A also has an N-terminal BAR (Bin/Amphiphysin/Rvs-homology) domain, formed by a three-helix bundle [8], [9], [10]. The dimeric BAR domain is crescent-shaped and can bind lipid membranes to remodel their structure, a crucial feature of endocytosis [11]. While the bending mechanism is debated [12], the molecular structure of the endophilin-A BAR domain is well documented [8], [9], [10], [13]. Endophilin-A BAR belongs to the N-BAR family, implying that it contains an N-terminal amphipathic helix, termed Helix 0, which folds upon membrane binding and partially embeds into the lipid bilayer [8], [9], [14]. A distinct feature of endophilin-A BAR is a central helix-loop appendage (also termed the Helix 1 insert) that protrudes from the concave surface of the crescent [8], [9], [10]. The helical component of the appendage forms a hydrophobic ridge that runs across the central concavity. The ridge is thought to assist the BAR domain in bending lipid bilayers [8].

The structural features of endophilin-A BAR that are functionally important have been defined mainly through assessment of curvature induction in vitro. Especially, the ability of wild type or mutant BAR domains to convert liposomes into tubules or vesicle-like structures has been documented [8], [9], [14]. In a biologically more realistic setting, the ability of overexpressed endophilin-A BAR to tubulate the plasma membrane in cultured cells has also been evaluated [8]. However, the “spaghetti-like” tubular networks appearing in these assays do not occur during physiological endophilin activity. Hence, despite the important information obtained, it is difficult to know to what extent tubulation in vitro and even in living cells gauge the normal function of endophilin. For example, does the missing ability of some endophilin-A BAR mutants to tubulate membranes predict loss of synaptic vesicle retrieval at the “behaving” synapse? To answer such questions and gain more insight into the in vivo function of endophilin-A BAR, we adopted a mutant rescue approach in *Drosophila*. By assessing a variety of physiological parameters, we studied to what extent endophilin-A transgenes that carry mutations in the N-BAR domain are able to compensate for lack of the endogenous endophilin-A gene (*endoA*). Although we observe a correlation between the severity of the mutational effects in vitro and in vivo, we also detect a number of notable exceptions to this rule. Moreover, we find that the *A66W* mutation has a unique, severely negative impact on development, which may be explained by enhancement of tubulation and vesiculation induced by this mutation in vitro [8].

## Materials and Methods

### Mutagenesis

The cDNA clone GH12907 containing the *endoA* coding sequence was obtained from the Drosophila Genomics Research Center (DGRC). The consensus sequence for EndoA, reported in Flybase, has lysine at position 129, whereas GH12907 has arginine. This likely reflects a polymorphism, since arginine and lysine have similar physicochemical properties. However, to comply with the consensus sequence, we modified GH12907 to encode 129R rather than 129K. This and subsequent site-directed mutagenesis was carried out using either the QuikChange kit (Stratagene, La Jolla, CA, USA) or a PCR amplification-based method (http://openwetware.org/wiki/Round-the-horn_site-directed_mutagenesis).

### Chimeras

**Figure 1:**
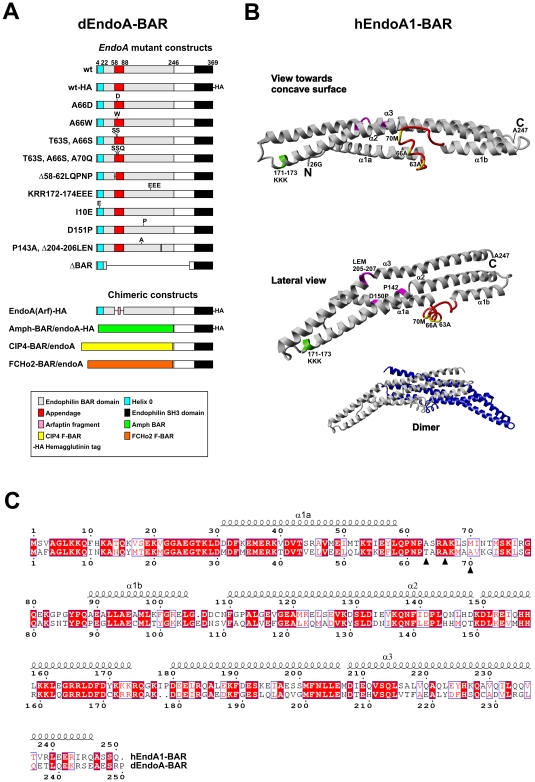
Targeted mutations in dEndoA-BAR and their relationship to the structure of hEndoA1-BAR. A, Schematic representation of the mutations introduced in the rescue constructs encoding dEndoA-BAR. B, Mutations homologous to the mutations in dEndoA-BAR (A), mapped onto the tertiary structure of hEndoA1-BAR monomer [PDB code 1X03A, 8]. The central helix-loop appendage (*red*) and the residues constituting the hydrophobic ridge (*yellow*) are indicated. The residues mutated to change the BAR domain curvature are also indicated (*pink*), as are the three electropositive lysine residues that were mutated to electronegative glutamic acid residues (*light green*). The *inset* at the lower right shows the BAR dimer, with the two monomers colored *gray* and *blue*. C, Primary structure alignment of hEndoA1-BAR (accession BAE44459.1; *top*) and dEndoA-BAR (accession CAD24682.1; *bottom*). The alpha-helical secondary structure is indicated by *squiggles*, based on the hEndoA1-BAR structure. The residues associated with the hydrophobic ridge are also indicated (*closed triangles*).

Overlap extension PCR was used to produce the four chimeras analyzed in this study (Figure 1A, bottom). To establish the FCHo2-BAR/endoA chimera, we performed a BLAST search of the *Drosophila melanogaster* genome, using the human FCHo2 F-BAR domain sequence as query. This identified the *CG8176* gene as the likely fly orthologue of FCHo2. The CG8176 polypeptide has four isoforms, A–D (Flybase annotation). The F-BAR domain in CG8176-PA and CG8176-PC is 44% identical with the F-BAR domain of human FCHo2. The FCHo2-BAR/endoA chimera consisted of the N-terminal 269 residues of CG8176-PA, fused to the C-terminal 125 AA of EndoA. As the template for PCR amplification of CG8176 F-BAR, we used the AT02057 cDNA clone obtained from DGRC. The CIP4-BAR/endoA chimera consisted of the N-terminal 289 AA of human CIP4, fused to the C-terminal 125 AA of EndoA. As template for PCR amplification of CIP4 F-BAR, the cDNA clone IRAUp969E1249D was used (ImaGenes, Berlin, Germany). The Amph-BAR/endoA chimera consisted of the N-terminal 238 AA of *Drosophila* amphiphysin containing the BAR domain, fused to the C-terminal 125 AA of EndoA. As template for PCR amplification of the Amph BAR domain sequence, we used the cDNA clone LD19810 (DGRC). In the EndoA(Arf) chimera, the central appendage in EndoA (AA 59–88) was deleted and replaced with a sequence (AHLSSLLQ) derived from the central concavity of the human arfaptin 2 BAR dimer [8], [15].

The *endoA* constructs were PCR amplified using primers with a 5′ tail containing *BglII* and *KpnI* sites (*Not1* and *KpnI* in the case of Amph-BAR/endoA-HA), for directional cloning into the pUAST transformation vector [16]. Fly transformation through pUAST injection into *w^1118^* embryos was carried out by VANEDIS (Oslo, Norway) or BestGene Inc. (Chino Hills, Ca, USA). Generally, at least two independent integration lines were tested for each of the *endoA* constructs.

### Drosophila Strains and Genetics

To assess the ability of UAS-endoA* transgenes to rescue *endoA* nulls, *w*; P{w^+^ UAS-endoA*} virgins were crossed to *+/Y*; *+/In(2LR)Gla*; *+/TM6B P{w^+^ Ubi-GFP.S65T}PAD2 Tb* males. F1 *w/Y*; P{w^+^ UAS-endoA*}/*In(2LR)Gla*; *+/TM6B P{w^+^ Ubi-GFP.S65T}PAD2 Tb* males were crossed to *w*; *+/CyO*; *endoA^Δ4^/TM3 P*{*w+ GAL4-Kr.C*}*DC2*, *P{w^+^ UAS-GFP.S65T}DC10 Sb* virgins. F2 *w/w* or *Y*; P{w^+^ UAS-endoA*}/*CyO*; *endoA^Δ4^/TM6B P{w^+^ Ubi-GFP.S65T}PAD2 Tb* progeny was crossed *inter se*, and *w/w* or *Y*; P{w^+^ UAS-endoA*}/P{w^+^ UAS-endoA*}; *endoA^Δ4^*/*TM6B P{w^+^ Ubi-GFP.S65T}PAD2* flies were used to generate a stock. In the final “rescue cross”, males from this stock were crossed to *P{GawB}elav^155^*; *+/+* ; *endoA^Δ4^/TM3 P{w+ GAL4-Kr.C}DC2*, *P{w+ UAS-GFP.S65T}DC10*, *Sb* virgins, to test for the presence of viable progeny of the genotype *w* or *Y*/*P{GawB}elav^155^*; +/P{w^+^ UAS-endoA*}; *endoA^Δ^* ^4^/*endoA^Δ^* ^4^ (this genotype is henceforth termed “rescuants”). Our initial crossing strategy involved the maintenance of both UAS-endoA* and the *TM3 GAL4-Kr UAS-GFP Sb* balancer in the same flies. This proved to be troublesome, possibly because the GAL4 produced under control of the *Kr* (*Krüppel*) promoter drove expression of the endoA* transgene, in addition to GFP expression. Instead, we combined UAS-endoA* with another third chromosome balancer *TM6B Ubi-GFP Tb*, leading to the crossing scheme presented above. Flies carrying the *endoA^Δ4^* allele [5] were kindly provided by H. Bellen. This allele was originally known as *endo^Δ4^* but is renamed here according to the current terminology adopted by Flybase to distinguish between the *endoA* and *endoB* genes [17]. All other stocks were obtained from the Bloomington stock center (http://flystocks.bio.indiana.edu/).

**Figure 2:**
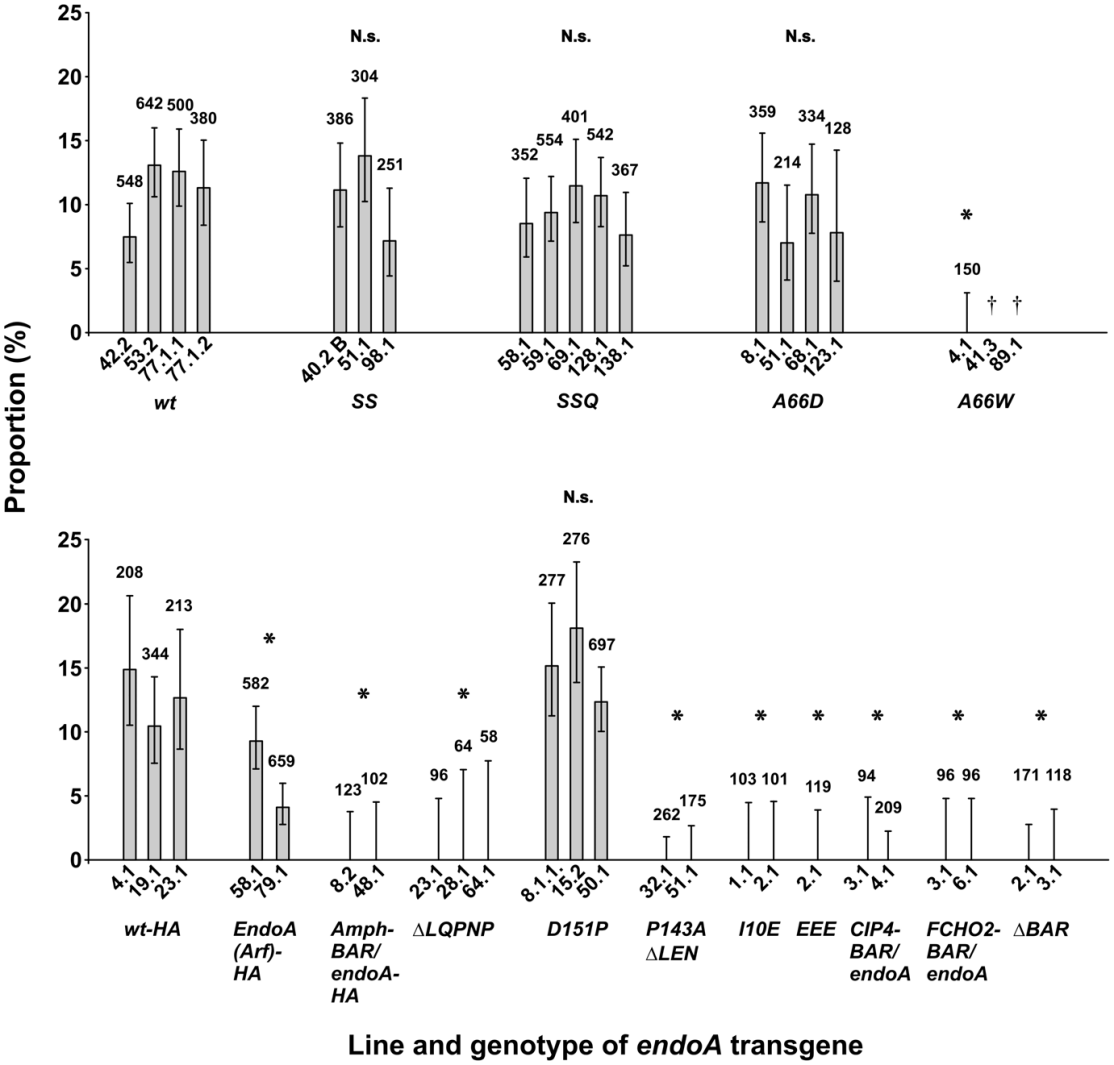
Ability of transgenic *endoA* constructs to rescue the development of *endoA* null mutants to adulthood. Shown is the proportion of eclosed adult rescuants (genotype *elav-GAL4/Y or w*; UAS-endoA*/+; endoA^Δ4^/endoA^Δ4^) relative to the total number of adult progeny resulting from the rescue cross. The UAS-endoA* transgene carried the mutations indicated on the abscissa and in some cases also encoded a hemagglutinin epitope tag (indicated by the suffix “−HA”). Also shown is the proportion of rescuants in which the *endoA* transgene encoded either wild type EndoA (“wt”), or HA-tagged wild type EndoA (“wt-HA”). Each *bar* represents one transgenic integration line, specified *below the abscissa*. The total number of adult progeny resulting from the rescue cross is indicated for each line (*numbers above the bars*). The lower and upper 95% confidence intervals are given. ^*^ *P*<0.01. N.s., not significant. ^†^Besides *UAS-endoA^A66W 4.1^*, the rescue efficiency of two other *UAS-endoA^A66W^* transgenes was evaluated (*UAS-endoA^A66W 41.3^* and *UAS-endoA^89.1^*). They both caused lethality of all the progeny from the rescue cross, as detailed in the text.

To calculate the proportion of EndoA nulls rescued by expression of UAS- endoA* (Figure 2), we divided the number of *elav-GAL4/w or Y*; UAS-endoA*/+; *endoA^Δ4^/endoA^Δ4^* adult rescuants with the total number of adult offspring from the rescue cross, having the four possible genotypes *elav-GAL4/w or Y*; UAS-endoA*/+; *endoA^Δ4^/endoA^Δ4^* or *elav-GAL4/w or Y*; UAS- endoA*/+; *endoA^Δ4^/TM3 Sb Kr-GFP* or *elav-GAL4/w or Y*; UAS-endoA*/+; *endoA^Δ4^/TM6 Tb Ubi-GFP* or *elav-GAL4/w or Y*; UAS-endoA*/+; *TM3 Sb Kr-GFP/TM6 Tb Ubi-GFP*.

To test for the effect of overexpressing endoA* in the CNS, second or third chromosome P{w^+^ UAS-endoA*} male homozygotes were crossed to *P{GawB}elav^155^* homozygous virgins, to produce *P{GawB}elav^155^/+ or Y*; P{w^+^ UAS-endoA*}/+ or *P{GawB}elav^155^/+ or Y;+/+*; P{w^+^ UAS-endoA*}/+ progeny.

To test the expression capability of UAS-endoA* transgenes in embryos, P{w^+^ UAS-endoA*} male homozygotes were crossed to *y w*; *P{w^+^ en2.4-GAL4}e16 P{w^+^ UAS-FLP1.D}JD1* homozygous virgins, to generate progeny with the only possible genotype P{w^+^ UAS-endoA*}/*y w* or *Y*; *P{w^+^ en2.4-GAL4}e16 P{w^+^ UAS-FLP1.D}JD1*.

### Western Blotting

Late-stage embryos were collected and homogenized in 1× Laemmli buffer containing 5% β-mercaptoethanol. The extracts were boiled for 5 min and cooled on ice, and a volume equivalent to 12 embryos per lane was separated on a 10% SDS-PAGE. The proteins were blotted on nitrocellulose and the blot was processed with chemiluminescent detection using the WesternBreeze kit (Invitrogen), following the manufacturer's protocol except that blocking was done overnight at 4°C. Primary antibodies were guinea pig anti-Endophilin (kind gift from H. Bellen, Houston) 1∶500, mouse anti-Elav (9F8A9, Developmental studies Hybridoma bank) 1∶1000 and rat anti-HA (3F10, Roche) 1∶1000. Secondary antibodies were taken from the WesternBreeze kit for mouse primary antibodies, to which was added 1∶10000 anti-Guinea pig-AP or 1∶20000 anti-rat-AP (both from Sigma). A similar procedure was employed for adult heads.

### Electrophysiology

Third instar larval fillets were prepared in an ice-cold extracellular solution containing (in mM): NaCl, 110; KCl, 5; NaHCO_3_, 10; trehalose, 5; sucrose, 30; HEPES, 5; MgCl_2_, 5; CaCl_2_, 5; pH 7.3. This solution was also used for intramuscular recordings. At the end of the dissection, the motor nerves innervating the body wall musculature were transected close to their exit from the ventral nerve cord. The preparation was transferred to the recording chamber and left there for at least 15 minutes before starting to record from the muscles. The temperature of the recording chamber was kept at 20°C using a TC-202A temperature controller (Harvard Apparatus, Holliston, MA, USA). Muscles were impaled with pulled thin-walled borosilicate glass capillaries (GC150TF-7.5, Harvard Apparatus) filled with a 3∶1 mixture of 4 M K-acetate and 3 M KCl. Current-clamp recordings were carried out in bridge mode in body wall muscles 6 and 7 in the A3–A5 segments of third instar larvae, using a 700A Multiclamp amplifier (Molecular Devices, Sunnyvale, CA, USA). Only recordings with a resting membrane potential more hyperpolarized than −60 mV were included in the data analysis. Recordings in which the membrane potential depolarized more than 10 mV during the recording session were excluded from analysis. Spontaneous miniature excitatory junctional potentials (mEJPs) were recorded continuously for 2 min and analyzed using the template search option in the Clampfit 9 program (Molecular Devices). To evoke excitatory junctional potentials (EJPs) in the muscles, the nerve was cut, placed in a suction electrode made of a glass capillary pulled in multiple steps and fire-polished, and stimulated with an A365 stimulus isolator (WPI, Sarasota, FL, USA).

### Locomotor Activity and Survival

Newly eclosed flies were aged singly for one day in small vials containing standard food. They were then transferred without anesthesia to a plastic box (10×10×1 cm), and the time they spent walking during a 60s observation period was measured under standardized illumination. Flies that were not able to stand after the transfer to the dish were helped to their feet once. To generate survival curves, eclosed flies were kept individually in small vials with standard food and checked for viability every morning.

### Statistical Analysis

Data were analyzed in a spreadsheet or using STATISTICA software (Statsoft, Tulsa, OK, USA). The level of significance was set to 5%. Confidence intervals (CIs) for proportions were calculated according to Fleiss [18] and Zar [19]. To evaluate differences in the proportions of rescuants among different genotypes (Figure 2), we followed a procedure analogous to the Dunnett test [18], [19]. The proportions were first subjected to angular transformation. Then, for each BAR mutation, the proportions for the different integrations were pooled. Finally, the pooled mutant proportions were each compared to the proportions of a control group in which the untagged and the HA-tagged wild type versions were pooled. In electrophysiological experiments, dual recordings were generally made from the same larva. Hence, the data were first analyzed using a two-factor nested ANOVA, with “larva” set to be a random factor nested into the second factor, “genotype”. However, since the effect of the nested factor was found to be insignificant, the data were re-analyzed using a single-factor ANOVA. In the behavioral assay (assessing the locomotor activity of adult flies), only one measurement was made on individual flies. However, the same genotype was generally represented by several UAS-endoA* integrations (lines). Therefore, these data were also analyzed using a two-factor nested ANOVA, setting “line” as a random factor nested into the second factor, “genotype”. When appropriate, data were square root-transformed or logarithmically transformed to comply with the assumptions underlying the ANOVA analysis. Analysis of survival of adult flies was performed according to Altman [20].

### Microscopy and Image Processing

#### Scanning electron microscopy

Flies were fixed in 2% glutaraldehyde in 0.05 M sodium phosphate buffer, pH 7.4. Following 3 rinses in 0.15 M sodium cacodylate buffer (pH 7.4) they were post-fixed in 1% OsO4 in 0.12 M sodium cacodylate buffer (pH 7.4) for 2 h. After a rinse in distilled water, the specimens were dehydrated to 100% ethanol according to standard procedures and critical point dried (Balzers CPD 030) employing CO2. Subsequently, they were mounted on stubs using colloidal coal as an adhesive, and sputter coated with gold (Polaron SEM Coating Unit E5000). Specimens were examined with a Philips FEG30 scanning electron microscope operated at an accelerating voltage of 1–5 kV.

Stereomicrographs of eyes were acquired with a Leica MZFLIII microscope. The primary structure alignment (Figure 1C) was decorated using the ESPript program by P. Gouet and F. Metoz [[21], http://espript.ibcp.fr]. The tertiary structure of hEndoA-BAR (Figure 1B) was rendered from data deposited in the Protein Data Bank (1X03A; Masuda et al. 2006), using the YASARA software (E. Krieger; YASARA Biosciences program, Graz, Austria; www.yasara.org). Images were digitally processed in CorelDRAW and Corel PHOTO-PAINT (Corel Corporation).

## Results

### Experimental Strategy

Our starting point was *endoA^Δ4^*, an *endoA* null allele created by imprecise excision of a P-element in *Drosophila* [5]. Normally, *endoA^Δ4^* homozygotes die as second instar larvae. However, using the modular UAS/GAL4 system [16], they can be rescued by nervous system expression of an *endoA* transgene, *UAS-endoA*, driven by the pan-neuronal transcription initiation protein *elav*-GAL4. The rescuants have the genotype *elav-GAL4/w or Y*; UAS-endoA*/+; *endoA^Δ4^/endoA^Δ4^*, where the generic term UAS-endoA* designates any of the mutant transgenes analyzed in this study, or controls without mutations. The key principle of this “mutant rescue” paradigm is that the rescue provided by UAS-endoA* is impeded to the extent that the mutations carried by UAS-endoA* interfere negatively with endophilin function.

### Rescue of EndoA Null Mutant Development

#### Point mutations in the hydrophobic ridge

Our mutational strategy was based on the solved tertiary structure of the *Homo sapiens* endophilin-A1 BAR domain [hEndoA1–BAR; [8]]. In hEndoA1-BAR, the central helix-loop appendage establishes a hydrophobic ridge that consists of alanines in position 63 and 66 and methionine in position 70 (Figure 1B). The hydrophobic ridge assists the bending of lipid membranes in vitro. Substituting Ala-66 with aspartate (*A66D*) that carries a membrane-repulsive negative charge disrupts the ability of hEndoA1-BAR to tubulate liposomes. Moreover, the hydrophilic substitutions *SS* (*A63S/A66S*) and *SSQ* (*A63S/A66S/M70Q*) strongly reduce the number of tubules and increase their diameter, reflecting a reduction in membrane bending [8]. Other point mutations affecting the ridge region have similar effects [9].

In the *Drosophila* Endophilin-A BAR domain (hereafter dEndoA-BAR or EndoA-BAR), the residues homologous to those forming the hydrophobic ridge in hEndoA1-BAR are also hydrophobic in two of the positions (alanine, positions 66 and 70), whereas he third residue is neutral (threonine, position 63; Figure 1C). Thus, a hydrophobic ridge organized exactly like in hEndoA1-BAR may not to be present in dEndoA-BAR. However, the hydrophobic residues at position 66 and 70 are highly conserved, and the region homologous to the ridge could still play an important role also in the fly protein. We therefore analyzed the rescue of *endoA* nulls obtained with *endoA* transgenes carrying the mutations *SS* (T63S/A66S), *SSQ* (T63S/A66S/A70S), or *A66D* (Figure 1A and 2). To quantify the rescue efficiency of these transgenes, we calculated the proportion of adult rescuants capable of eclosing (see Methods). Three to five transgenic integrations were evaluated for each *UAS-endoA* mutation. To our surprise, *UAS*-*endoA^SS^*, *UAS*-*endoA^SSQ^*, and *UAS*-*endoA^A66D^* expression driven by *elav*-GAL4 rescued *endoA* nulls to adulthood with the same efficiency as the wild type transgene *UAS*-*endoA^wt^* (Figure 2, top). These results demonstrate that mutations in the central appendage, which disrupt curvature induction mediated by hEndoA1-BAR in vitro, do not necessarily impede the in vivo functions of dEndoA-BAR.

#### Rescue with UAS-endoA^A66W^

Substituting Ala-66 in the hydrophobic ridge with the bulky hydrophobic residue tryptophan (*A66W*; Figure 1A) leads to extensive vesiculation in the liposome assay [8]. Rescuants carrying either of two integrations of a transgene with this mutation, *UAS-endoA^A66W 41.3^* or *UAS-endoA^A66W 89.1^*, died as embryos or pupae, respectively. A third integration, *UAS*-*endoA^A66W 4.1^*, was also unable to rescue the development of *endoA* nulls to adult flies capable of eclosing (Figure 2). However, development of these flies proceeded as far as pharate adults.

#### UAS-endoA^A66W^ overexpression

Referring to the previous paragraph, not just the *endoA* nulls but all progeny from the rescue crosses that involved *UAS-endoA^A66W 41.3^* or *UAS-endoA^A66W 89.1^* died early. An obvious explanation is that all the progeny also carried the active combination of *elav-GAL4* and *UAS-endoA^A66W^* (see Methods). To further explore this issue, we also analyzed “simple” *elav-GAL4*-driven overexpression of the *UAS-endoA^A66W^* transgene on a wild type *endoA* background, in flies carrying the *UAS-endoA^A66W^* integration either on the second or the third chromosome. Simple overexpression of *UAS-endoA^A66W^* (*UAS-endoA^A66W 4.1^* not included, see below) was lethal either at the embryonic stage (6 integrations) or at the pupal stage (9 integrations). By contrast, *UAS-endoA^A66W 4.1^* overexpression did not prevent development into adulthood. However, the flies were weak, lived only a few days after eclosion, and could not inflate their wings. They also exhibited a distinct small-eye phenotype (see below). Viability and development was not affected by *elav*-Gal4-driven overexpression of any of the other UAS-endo* transgenes tested in this study.

#### A66W perturbs eye development

**Figure 3:**
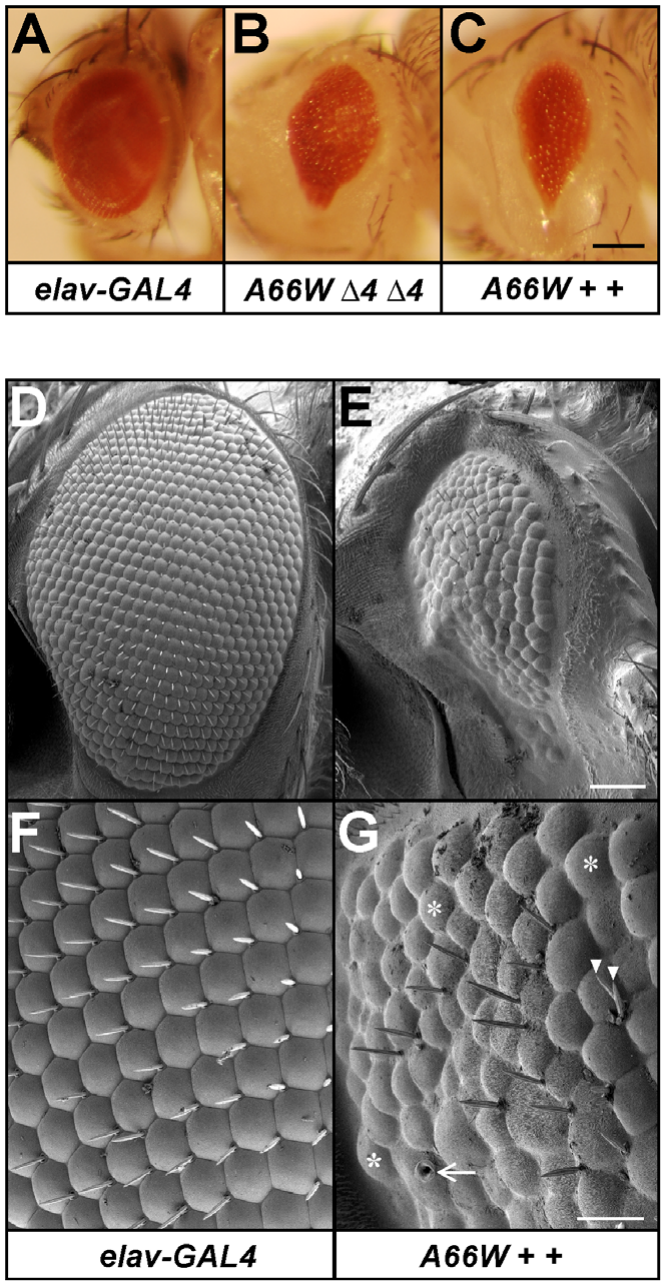
The small-eye trait induced by expression of the *UAS-endoA^A66W^* mutant transgene. A, eye of a control fly carrying the *elav-GAL4* driver but no *endoA* transgene. B, *endoA* null fly rescued to the pharate adult stage by *UAS-endoA^A66W^* expression, driven by *elav-GAL4* (*elav-GAL4/w*; *UAS-endoA^A66W 4.1^/+*; *endoA^Δ4^/endoA^Δ4^*). Note that the eye size is reduced and that the lower eye tip is pointy rather than rounded. C, The small-eye trait also appears when *UAS-endoA^A66W^* expression occurs on a wild-type *endoA* background (*elav-GAL4/w*; *UAS-endoA^A66W 4.1^/+*; *endoA^+^/endoA^+^*). D–G, Scanning electron micrographs of *elav-GAL4* (D, F) and *elav-GAL4/w*; *UAS-endoA^A66W 4.1^/+*; *endoA^+^/endoA^+^* (E, G) eyes. In G, some examples of ommatidia that lack bristles are indicated by *asterisks*, and aberrant dual bristles by *arrowheads*. Pitting is indicated by an *arrow*. Scale bars: C, 100 µm (applies to A–C); E, 50 µm (D, E); G, 20 µm (F, G).

We found that *UAS-endoA^A66W 4.1^* expression driven by *elav-GAL4* leads to the formation of small eyes, both in *endoA* null rescuants and in flies carrying the normal dose of endogenous wild type *endoA* (that is, overexpressing *UAS-endoA^A66W 4.1^*). In addition to the reduced eye size, the lower aspect of the eye was typically narrow and pointed rather than having the usual rounded contour (Figure 3B,C). Moreover, scanning electron micrographs revealed roughening of the eye surface. The ommatidia were uneven in size, and the bristles were often missing or supernumerary (Figure 3E,G). Moreover, there was abnormal “pitting” in the ommatidia, suggesting defects in the cone cells that secrete the lens material [22; Figure 3G, arrow].

Similar small eyes were also observed in late pupae that overexpressed other *UAS-endoA^A66W^* integrations than *UAS-endoA^A66W 4.1^* (not shown). By contrast, small eyes never occurred in flies carrying only *UAS-endoA^A66W^* without the *elav-GAL4* driver or those carrying the *elav-GAL4* driver alone. Also, whereas some integration lines of the *UAS-endoA^SS^* and *UAS-endoA^SSQ^* rescuants displayed a tendency for malformation of the wings, they never showed the small eye trait. In general, none of the other UAS-endoA* transgenes investigated in our study had effects nearly as severe as *UAS-endoA^A66W^*. In conclusion, the *A66W* mutation has a unique, strongly negative impact on development and viability, also in the presence of the normal dose of endogenous *endoA^+^*.

#### Larger mutations targeting the central appendage

In hEndoA1-BAR, the N-terminal aspect of the appendage includes a highly conserved stretch of five residues (58-62LQPNP; Figure 1C). We found that *UAS*-*endoA^ΔLQPNP^*, in which the homologous five residues of dEndoA-BAR were deleted (Figure 1A), failed to rescue the development of *endoA* nulls to adulthood (Figure 2). It is possible that the *ΔLQPNP* deletion disrupts dEndoA-BAR folding or dimerization (see below). Therefore, we also tested *UAS*-*endoA^Amph-BAR/endoA^*, in which the N-BAR domain of EndoA is replaced with the N-BAR domain of *Drosophila* amphiphysin (Figure 1A). The idea behind this experiments is that the tertiary structure of the crescent scaffold is likely to be similar for the EndoA and Amph N-BAR domain [9], [11], whereas the Amph-BAR is devoid of the central helix-loop appendage present in the EndoA-BAR. We found that the *UAS*-*endoA^Amph-BAR/endoA^* transgene was unable to rescue the *endoA* null mutants (Figure 2). Finally, we tested the rescuing capability of *UAS*-*endoA^EndoA(Arf)-HA^*, in which the entire helix-loop appendage is replaced by AHLSSLLQ, a helical stretch derived from the sequence of human arfaptin 2 (Figure 1A). The homologous mutation in hEndoA1, *ΔApp*, impedes liposome tubulation and increases the diameter of the tubules [8]. Interestingly, *UAS*-*endoA^EndoA(Arf)-HA^* rescued the development of *endoA* null mutants to adulthood, although the rescue was somewhat inferior to that provided by the wild type control transgenes (Figure 2). We conclude that loss of the central helix-loop appendage of dEndoA-BAR impedes the development into adults to some extent. However, substantial in vivo function of endophilin-A is retained even after total ablation of the appendage.

#### Mutations targeting the dEndoA-BAR curvature

As a first step towards judging the importance of the exact dEndoA-BAR curvature in vivo, we made mutations designed to induce curvature changes. In dEndoA-BAR, the proline at position 143 (Pro 142 in hEndoA1-BAR) produces a kink in the α2 helix, which contributes to the crescent shape of the dimer (Figure 1). To increase the curvature of dEndoA-BAR, we mutated alanine to proline at position 151 in order to add an additional kink in the same plane as the kink produced by Pro 143. The resulting Pro 151 is situated exactly two helix turns away from Pro 143. This transgene, *UAS*-*endoA^D151P^*, rescued *endoA* null mutants to adulthood with the same efficiency as the wild type transgene *UAS*-*endoA^wt^* (Figure 2).

Conversely, to stretch the dEndoA-BAR domain, we changed the proline responsible for the kink in the wild type protein to alanine (P143A) and shortened helix α3 by deleting the three residues LEN 204–206 (the homologous mutations in hEndoA1-BAR are P142A and ΔLEM 205–207; Figure 1A and B). The resulting transgene, *UAS*-*endoA^P143A, ΔLEN^*, was unable to rescue the *endoA* null mutants (Figure 2).

These results are compatible with the notion that a moderate increase in the bending of the dEndo-BAR domain does not perturb its function. By contrast, stretching the dEndo-BAR domain may be less tolerable.

#### Negatively charged mutations outside the central appendage

Helix 0 is the N-terminal amphipathic helix that classifies the endophilin BAR domain as a N-BAR family member. Helix 0 contributes importantly to the in vitro function of the endophilin BAR domain [8], [9], [23]. It is formed by the residues ∼4–22 in endophilin-A 1–3. Within Helix 0, a hydrophobic residue is conserved at position 10. This residue is phenylalanine in the mammalian endophilin-A isoforms, and isoleucine in *Drosophila* EndoA. The *F10E* mutation, which in mammals replaces phenylalanine with the negatively charged glutamate, reduces both liposome binding and tubulation [9] and also disrupts curvature sensing [23]. In accordance with this result, we found that the transgene carrying the homologous mutation in *Drosophila*, *I10E* (Figure 1A), could not rescue the development of the *endoA* nulls (Figure 2).

Electropositive patches at the concave BAR dimer surface are strongly implicated in membrane binding in vitro. To test the effect of converting electropositive side chains at one such patch to membrane-repulsive negative ones, we introduced three sequential glutamate residues (*EEE*: K172E/R173E/R174E) near the ends of the dEndoA-BAR dimer (Figure 1A) [9], [11]. We found that *UAS-endoA^EEE^* failed to rescue the *endoA* nulls (Figure 2). To test if Helix 0 on its own might be sufficient to mediate some rudimentary function of the N-BAR, we deleted most of the BAR domain, leaving essentially only Helix 0 and the SH3 domain (Figure 1A). This transgene (*UAS-endoA^ΔBAR^*) also could not rescue the *endoA* null mutants (Figure 2).

These results corroborate work in reduced systems, that both the N-terminal amphipathic helix and the electropositive patches at the BAR concavity strongly contribute to the function of the endophilin N-BAR domain.

#### Rescue constructs involving the entire N-BAR domain

Is an N-BAR domain strictly required for endophilin-A function in vivo, or can it be replaced by a BAR domain of a different family? F-BAR domains form crescent-shaped dimers that are larger and bend less than the N-BAR domain [24], [25]. Like the N-BAR, the F-BAR has powerful membrane-tubulating activity [24], [26], [27]. We produced chimeras fusing the F-BAR domain of either the human CIP4 or the fly FCHo2 protein to the linker region and SH3 domain of endophilin (Figure 1A). None of the corresponding transgenes *UAS-endoA^CIP4-BAR/endoA^* or *UAS-endoA^FCHo2-BAR/endoA^* could rescue the *endoA* nulls (Figure 2). Thus, an N-BAR domain appears to be indispensable for the in vivo function of endophilin.

### Expression of Rescue Constructs

**Figure 4:**
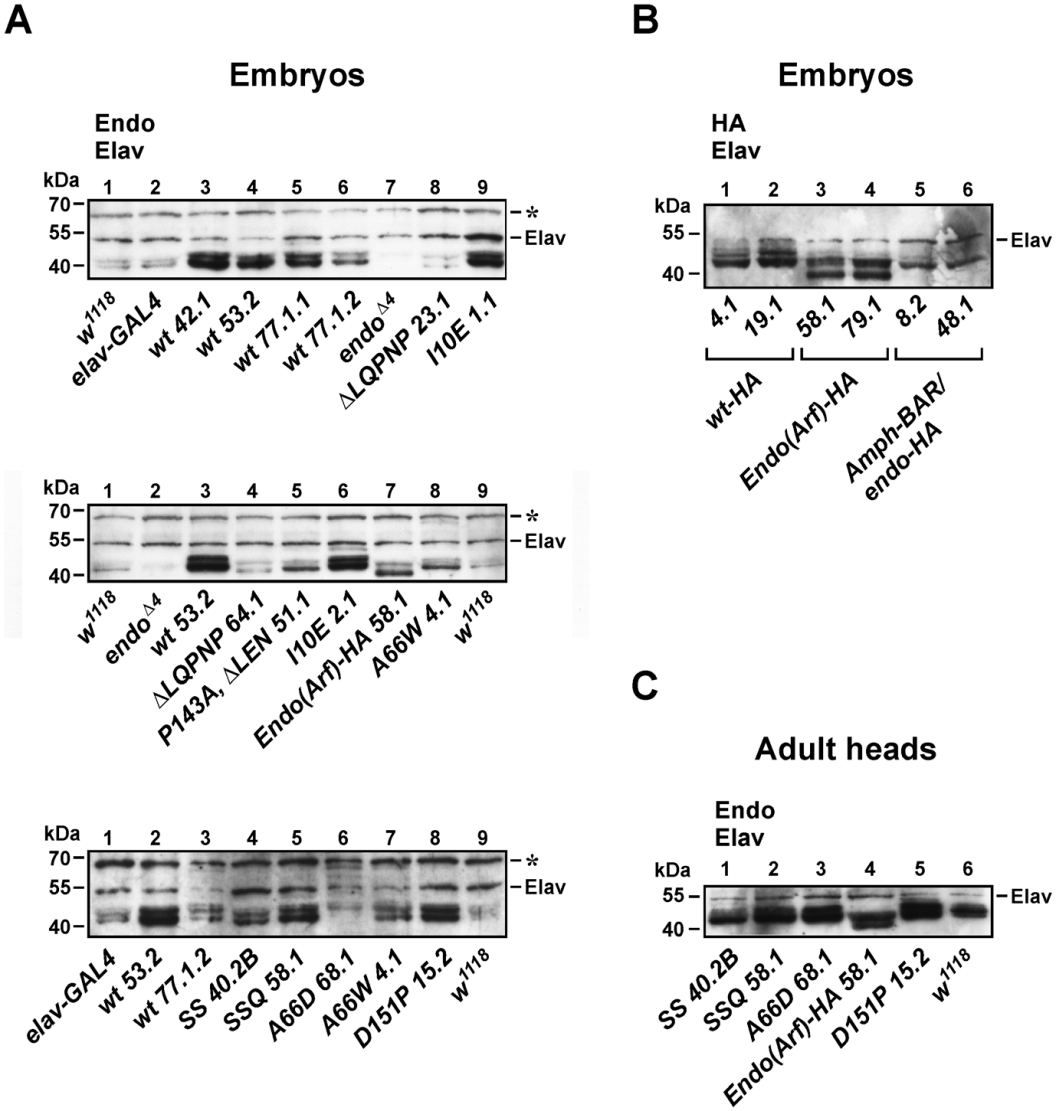
Expression capability of UAS-endoA* transgenes. A, Western blots of extracts from late-stage embryos, probed simultaneously with anti-EndoA and anti-Elav primary antibodies. Shown are genotypes without transgenes (*w^1118^* and *elav-GAL4*), *EndoA^Δ4^* null mutants, and null mutants carrying the indicated *endoA* transgenes driven by *elav-GAL4*. Note that some genotypes appear more than once. The EndoA immunosignal (wild type or mutant) generally runs as a doublet with the lower band matching the predicted size of EndoA (41.4 kDa). The signals from Elav and an unidentified protein (*asterisk*) both serve as loading controls. The *UAS-endoA^EndoA(Arf)-HA^* product runs distinctly lower than other products, due to the deletion of the entire BAR appendage. B, Extracts from null mutants carrying the indicated HA-tagged *endoA* transgenes driven by *elav-GAL4*, probed simultaneously with anti-HA and anti-Elav. C, Extracts from fly heads of EndoA nulls, rescued to adulthood with mutant EndoA transgenes (“successful transgenes”) and probed with anti-EndoA and anti-Elav. Numbers above lanes in all panels are for reference only.

Above, we have only considered the possibility that the failure of some UAS-endoA* transgenes to rescue the development of the *endoA* nulls is due to the mutation carried by those transgenes. However, although the GAL4/UAS system generally produces robust expression, the expression level of UAS-transgenes may depend on the integration site of the carrier P-element transposon. To reduce the influence of this position effect, we generally tested multiple integration lines for the same mutant transgene (Figure 2). However, it was still pertinent to characterize the relation between the ability of the different transgenes to rescue *endoA* nulls, and their levels of expression. Therefore, Western blots were made of proteins extracted from late-stage embryos (Figure 4A and B). The endophilin immunosignal generally appeared as a doublet with the lower band running close to the predicted size of 41.4 kDa (wild type EndoA). The Elav immunosignal, running at about 50 kDa, was used as loading control. An unidentified protein, present also in *endoA^Δ4^* null mutants, served as a convenient supplementary loading control (Figure 4A, asterisk). We found GAL4-driven expression of transgenic wild type or mutant EndoA to be generally higher than the expression of endogenous EndoA (Figure 4A). Hence, the fact that none of our transgenes provided better rescue than to weak adults (see below) cannot be caused by insufficient EndoA expression, but probably relates to the inability of the UAS/*elav*-GAL4 system to precisely mimic the activity of the endogenous *endoA* promoter.

EndoA expression of wild type rescuants at the embryonic stage was similar in the three lines *UAS-endoA^wt 42.1^*, *UAS-endoA^wt 53.2^* and *UAS-endoA^wt 77.1.1^*, while being somewhat weaker in a fourth line, *UAS-endoA^wt 77.1.2^* (Figure 4A top, lanes 3–6 and bottom, lanes 2–3). *endoA* null flies rescued to adulthood by the mutant transgenes *UAS-endo^SS 40.2B^*, *UAS-endo^SSQ 58.1^*, *UAS-endo^A66D 68.1^* and *UAS-endo^D151P 15.2^* displayed embryonic expression levels that ranged between the level of the most weakly expressing wild type line *UAS-endoA^wt 77.1.2^* and the strong line *UAS-endoA^wt 53.2^* (Figure 4A bottom, lanes 2–6 and 8). The expression associated with *UAS-endoA^A66W 4.1^*, which rescued the *endoA* nulls to pharate adults, was roughly similar to that of the weak line *UAS-endoA^wt 77.1.2^* (Figure 4A bottom, compare lanes 3 and 7).

#### EndoA expression of unsuccessful transgenes

In general, insufficient EndoA expression did not provide an obvious explanation for the failure of some mutant *endoA* transgenes to rescue the *endoA* nulls. For example, a strong EndoA immunosignal (similar to that in the strongly expressing wild type rescuants) was observed in *UAS-endo^I10E^* (lines *1.1* and *2.1*, Figure 4 *top*, lane 9; and *middle*, lane 6, respectively) and in *UAS-endo^EEE 2.1^* (data not shown). Moreover, the EndoA expression of *UAS-endo^P143A,ΔLEN 51.1^* matched the expression of *UAS-endoA^wt 77.1.2^*. One exception to this notion is the immunosignal produced by the deletion mutant *UAS-endoA^ΔLQPNP^*, which was consistently found to be weaker than that of even the weakly expressing control *UAS-endoA^wt 77.1.2^* (Figure 4A *top*, lane 8 and *middle*, lane 4).

Blots of embryonic rescuants carrying HA-tagged transgenes revealed that the expression level of *UAS-endoA^EndoA(Arf)-HA^* lines was similar to the level of *UAS-endoA^wt-HA^* lines (Figure 4B, lanes 1–4; note that the *EndoA(Arf)-HA* signal runs lower than the *UAS-endoA^wt-HA^* signal due to the deletion of the central BAR appendage). The immunosignal of *UAS-endoA^Amph-BAR/endoA-HA^* was somewhat weaker than that of *UAS-endoA^wt-HA^* and *UAS-endoA^EndoA(Arf)-HA^* on some gels (Figure 4B, lanes 5–6). However, on others it matched the signal of the two other genotypes (not shown).

The epitope recognized by the anti-EndoA antibody is missing in the *UAS-endoA^CIP4-BAR/endoA^*, *UAS-endoA^FCHo2-BAR/endoA^*, and *UAS-endoA^ΔBAR^* transgenic products, which were also not HA-tagged. Hence, their expression levels were not assessed.

### Rescue of Neurotransmission

#### Tetanus-induced depression

**Figure 5:**
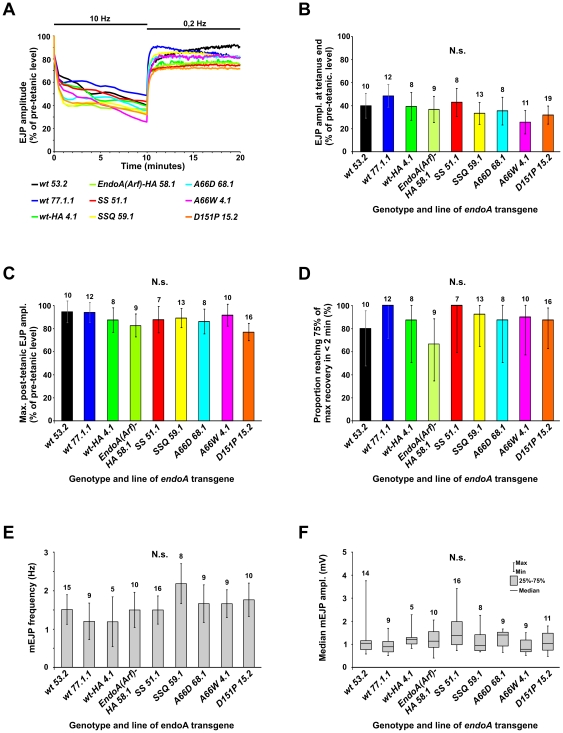
Neurotransmission at the neuromuscular junction in larval mutant rescuants. Intracellular recordings were made from the somatic muscles of *Elav-GAL4/Y or w*; UAS-endoA*/+; *endoA^Δ4^/endoA^Δ4^* third instar larvae, where UAS-endoA* represents a mutated *endoA* transgene, or one encoding wild type EndoA (“wt”), as indicated in A–F. The suffix “−HA” signifies the presence of an additional HA tag. Raw recordings are not shown. A, Ability to sustain neurotransmitter release during a tetanus (10 min at 10 Hz) and immediately following tetanic stimulation (10 min at 0.2 Hz). The amplitude of the excitatory junctional potential (EJP), relative to the amplitude prior to the tetanus (0.2 Hz, not shown), is plotted. Error bars are omitted in A for clarity; the variability can be judged from B–D. B, The EJP amplitude (mean and 95% confidence interval) at the end of the 10 Hz tetanus, just before switching back to stimulation at 0.2 Hz (*arrow* in A). C, The maximal EJP amplitude (mean and 95% confidence interval) observed in the 10 min post-tetanic recovery period. D, The proportion of cases, in which the EJP took less than two minutes to recover from the end-tetanic level (arrow in A) to at least 75% of the maximal post-tetanic EJP amplitude, after switching from 10 Hz to 0.2 Hz stimulation. The bars indicate 95% confidence intervals. E and F, Frequency and amplitude of miniature excitatory junctional potentials (mEJPs). For each genotype and line, *n* is shown above the bars. N.s., not significant.

The neuromuscular junction (NMJ) in larval body wall muscles is well suited for analysis of the synaptic function of EndoA [4], [17], [28], [29]. By assisting clathrin-dependent endocytosis of synaptic vesicles, EndoA contributes critically to the maintenance of synaptic transmission over long periods [4], [17], [28]. To analyze mutant rescue of prolonged synaptic vesicle retrieval, we performed intramuscular recordings of the excitatory junctional potential (EJP). First, a baseline EJP was obtained by stimulating the motor nerve at 0.2 Hz. Next, we stimulated at 10 Hz for 10 minutes (tetanic stimulation) and monitored the resulting changes over time in the evoked EJPs [4], [5]. We recorded from *endoA* nulls rescued to the third instar stage by expression of either the wild type transgenes *UAS-endoA^wt 53.2^*, *UAS-endoA^wt 77.1.1^* and *UAS-endoA^wt-HA 4.1^*, or the mutant transgenes *UAS-endoA^endoA(Arf)-HA 58.1^*, *UAS-endoA^SS 51.1^*, *UAS-endoA^SSQ 59.1^*, *UAS-endoA^A66D 68.1^*, *UAS-endoA^D151P 15.2^* and *UAS-endoA^A66W 4.1^*. In all these rescuants, a fast depression in the EJP amplitude occurred within the first minute of the tetanus, followed by a depression that developed more slowly (Figure 5A). Generally, the EJP amplitude at the end of the tetanus declined to ∼30–50% of the baseline amplitude. There was some tendency of the rescuants expressing wild-type *endoA* transgenes to withstand tetanic depression better than those expressing mutant transgenes (Figure 5A). However, when comparing all the rescuant genotypes, including both those expressing wild type and mutant transgenes, no significant differences were found among the means of their end-tetanic EJP, although the *P* value was not far from the critical level of 5% (ANOVA: *P* = 0.09; Figure 5B). Moreover, the tetanus-induced depression observed in both wild type and mutant rescuants was similar to the depression reported earlier in control larvae ( that is, non-rescuants, [4], [5]). Thereby, it was quite different from the severe depression seen in pure *endoA* null mutants, in which the EJP amplitude drops to 10–20% of the pre-tetanic EJP amplitude immediately after the onset of 10 Hz stimulation, and then remains at this low level throughout the tetanus [4], [5], [28].

#### Post-tetanic recovery

The EJP amplitude normally recovers almost fully from tetanic depression within a few seconds after returning to the basal stimulation frequency. In pure *endoA* nulls, recovery is much slower and not completed even after 10 minutes [4]. When we monitored the recovery of the rescuants in the first 10 minutes immediately after the tetanus (Figure 5A), we found that recovery of both wild type and mutant rescuants was robust and similar to the recovery reported in normal larvae [4]. We measured the maximal EJP amplitude seen in the post-tetanic period, expressed as the percentage of the pre-tetanic EJP (Figure 5C). The strongest recovery was observed in two of the three wild type lines (*UAS-endoA^wt 53.2^* and *UAS-endoA^wt 77.1.1^*; Figure 5A and C). Again, however, although the *P* value was not very far from 5%, no significant differences were found in the recovery among the nine rescuant lines tested (ANOVA: *P* = 0.10).

To evaluate the speed of recovery from tetanic stimulation, we calculated the proportion of cases in which the EJP took less than 2 minutes to reach 75% of the maximal EJP amplitude observed in the post-tetanic phase. This was done for each genotype, taking the end-tetanic EJP level as baseline. Generally, the resulting proportions were high (around 80% or more, Figure 5D) regardless of whether the rescuants expressed wild type or mutant *endoA* transgenes. This demonstrates a universal ability of the rescuant NMJs to recover quickly from tetanic depression.

#### Spontaneous vesicle release

The EJP frequency of spontaneous miniature EJPs (mEJPs) were found to be lower in *endoA* null mutant larvae than in controls [5]. By contrast, we neither detected differences in the mEJP frequency of the eight rescuant lines tested (Figure 5E; *P* = 0.31), nor between their mEJP amplitude (Figure 5F; *P* = 0.23).

### Differential Effects of *endoA* Mutations in Adult Flies

To summarize the above results, the various *endoA* transgenes generally either completely failed to rescue the *endoA* nulls, or enabled a significant proportion of them to develop into adulthood. Moreover, the transgenes that successfully rescued the *endoA* nulls also restored sustained neuromuscular transmission and normal patterns of spontaneous vesicle release at the larval NMJ, even when carrying mutations in the EndoA-BAR domain (Figure 5). We also noted that the behavior of the larvae, irrespective of whether they were rescued by mutant or wild type transgenes, did not differ notably from the behavior of wild type larvae (e.g., *w^1118^*). Specifically, we never observed the sluggish movements or the paralysis that characterize pure *endoA* null mutant larvae [5].

However, some aspects of the electrophysiological results (the tendency of wild type rescuants to perform slightly better than mutant rescuants, with “close-to-significance” outcome of some of the statistical tests) also hinted that perhaps in a different context the rescue provided by wild type *endoA* transgenes might prove to be superior to the rescue provided by the mutant transgenes. To investigate if we could demonstrate such overt effects of the mutations, we turned towards the adult rescuants.

#### Life span

**Figure 6:**
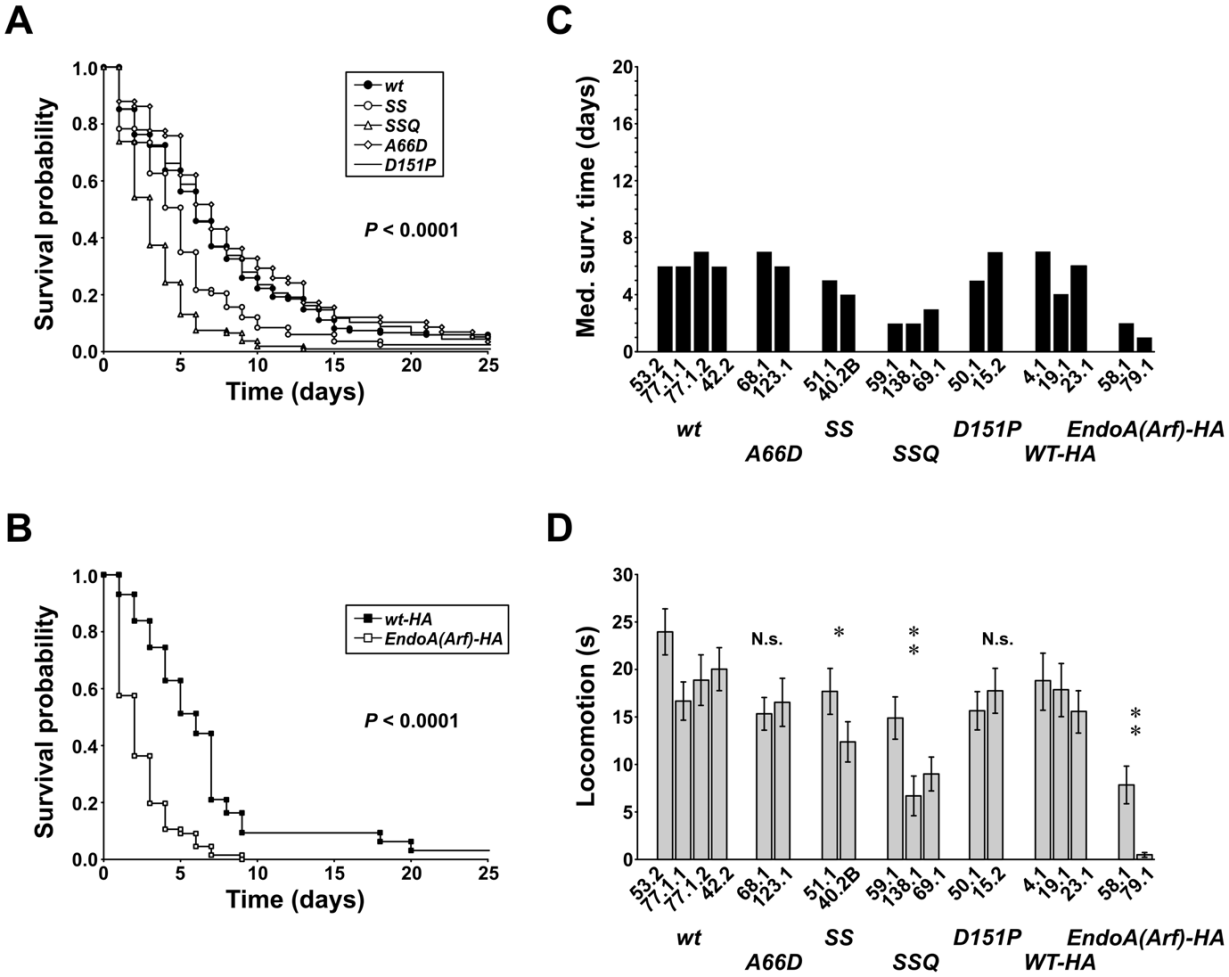
Life span and locomotor activity of adult rescuants. A, Kaplan-Meier survival curves showing the post-eclosion life span of the rescuants. The UAS-endoA* transgene either carried the mutations indicated in the Figure, or wild type *endoA* (“wt”). B, survival curves for the *EndoA(Arf)-HA* rescuants (*closed squares*) and control rescuants carrying an HA-tagged wild type *endoA* transgene (“wt-HA”, *open squares*). C, The median survival time (MST) of individual transgenic integration lines. On average, 32 flies per line were included in the survival analysis (range 12–52). D, The locomotor activity period (LAP; mean ± SEM). On average, 27 flies per line were included in the locomotion analysis (range 12–37). For statistical analysis, the rescue of EndoA(Arf)-HA was compared with the rescue provided by the HA-tagged *endoA^+^* transgene (“wt-HA”). Otherwise, the rescuants associated with the untagged *endoA^+^* transgene were used as controls (“wt”). ^*^ *P*<0.01, ^**^ *P*<10^−9^. N.s., not significant.

First, we constructed survival curves for adult *endoA* nulls, rescued with either wild type or successful mutant transgenes. Data from two to four transgenic integrations of the same genotype were pooled for each curve (Figure 6A and B). We also calculated the median survival time (MST) for individual lines (Figure 6C). The average MST of the *endoA* nulls rescued by wild type *endoA* (“wild type rescuants”) was 6.3 days, considerably lower than the MST reported for commonly used control genotypes, such as *w^1118^* [50–60 days; 30]. Clearly, the rescue provided even by the *endoA^wt^* transgene is only partial (also reflected in Figure 2, where the proportion of rescuants is always lower than the Mendelian ratio of 25%). However, the life span of the adult wild type rescuants was still long enough to allow a meaningful comparison with the rescue provided by the mutant transgenes. The survival of *endoA* nulls rescued by the wild type transgene (MST 6.3 days) was very similar to the survival obtained with the transgenes carrying *D151P* (MST 6.0 days) and *A66D* (MST 6.5 days). By contrast, the *SS* rescuants died faster (MST 4.5 days) and the *SSQ* rescuants considerably faster (MST 2.3 days) than the wild type rescuants (Figure 6A). This difference was highly significant (log-rank test for more than two groups, *P*<0.0001, Figure 6A). The shortest survival was seen in flies rescued with the *endoA(Arf)-HA* transgene (MST 1.5 days), clearly worse than the survival of flies rescued by a control transgene (*wt-HA*, MST 5.7 days; *P*<0.0001).

#### Locomotor activity

None of the *endoA* nulls rescued to adulthood could fly, but they were able to walk. However, the vigor of their locomotor activity depended on the mutation carried in the rescuing transgene. We counted the number of seconds in which the adult rescuants displayed locomotor activity when observed for one minute (locomotor activity period, LAP; Figure 6D). The average LAP in *A66D* and *D151P* rescuant lines was 15.9 s (range 15.3–16.5) and 16.8 s (15.7–17.8), respectively. This was lower than the LAP of wild type rescuants (19.9 s, 16.7–24.0), but the differences were not quite significant at the 5% level (*wt vs. A66D*, *P* = 0.09; *wt vs. D151P*, *P* = 0.10; Figure 6D). Furthermore, the locomotor activity of the *SS* rescuants was clearly reduced, and even more so in the *SSQ* rescuants. The LAP of these genotypes was 15.0 s (12.4–17.7) and 10.2 s (6.7–14.9), respectively; significantly lower than in wild type rescuants (*wt vs. SS*, *P*<0.01; *wt vs. SSQ*, *P* = 10^−9^). Hardly any locomotor activity was observed in flies rescued by the *EndoA(Arf)-HA* transgene. Their LAP amounted to only 4.2 s (0.5–7.8), much shorter than in the endoA-HA control rescuants (LAP 17.4 s, 15.5–18.7; *wt-HA vs. EndoA(Arf)-HA*, *P*<10^−5^; Figure 6D). Thus, mutating the dEndoA-BAR domain clearly had a negative impact on locomotor activity in adult rescuants, in contrast to the much weaker effect in larvae. Moreover, the severity of this effect depended on the mutations in a pattern similar to the pattern seen in the survival experiments.

#### Expression of transgenes in adult rescuants

Western blots of extracts from heads of adult rescuants revealed similar and robust expression levels for all the mutant transgenes discussed in the previous section, represented by the lines *UAS-endoA^SS 40.2B^*, *UAS-endoA^SSQ 58.1^*, *UAS-endoA^A66D 68.1^*, *UAS-endoA^D151P 15.2^* and *UAS-endoA^EndoA(Arf)-HA 58.1^* (Figure 4C).

In conclusion, mutations targeting the central helix-loop appendage on the dEndoA-BAR domain can severely depress survival and spontaneous motor activity of adult flies. This implies that the central appendage has an important functional role *in vivo*, in accordance with previous in vitro reports. By contrast, adult fly viability and locomotion were largely unaffected by the *D151P* mutation designed to increase the BAR domain curvature.

## Discussion

Here, we have assessed the structure/function relationship of the endophilin-A BAR domain in a physiological context, by testing the ability of mutated *endoA* transgenes to rescue the viability, locomotion and neurotransmission of *endoA* null mutants. While many of our results can be reconciled with conclusions drawn from earlier studies in reduced systems, there are some notable exceptions.

In the following, we will mainly focus on the structural aspects of the BAR domain and its interaction with the lipid membrane. This narrow scope is justified when treating those mutations that did not interfere negatively with transgenic rescue of the *endoA* nulls. However, for those mutations that prevented the rescue, it should be kept in mind that loss-of-function in vivo may also relate to other aspects. One such aspect is protein stability. Our Western blot results indicate that the mutant proteins do not in general suffer from low stability, with the possible exception of EndoA*^ΔLQPNP^*. Still, other possibilities remain, for example that compromised protein targeting to or within the synapse contributed to the loss-of-function of some transgenic alleles (see also the discussion of the F-BAR domain chimeras below).

### Role of Helix 0 and the Electropositive Patch

The *I10E* mutation replaces a conserved hydrophobic residue in the N-terminal amphipathic Helix 0 with a negatively charged one. We found that the *I10E* transgene could not rescue the development of the *EndoA* nulls. Failure of *F10E* endophilin to rescue *endophilin-A* mutants in *C. elegans* has also been reported [31, cited in Gallop et al. 2006]. Our results corroborate earlier biochemical work demonstrating the high functional importance of Helix 0. Specifically, in the liposome assay, *F10E* (the mutation in rat endophilin that is homologous to *I10E*) severely reduces lipid binding and disrupts tubulation [9], [14]. Moreover, single-liposome analysis indicates that the curvature-sensing ability of endophilin-A depends only on Helix 0 and is abolished by *F10E* [23].

The *EEE* mutation (*KRR172-174EEE*) reverses positive charges on a patch on the BAR domain concavity to negative charges. Like *F10E*, the homologous *EEE* mutation in mammalian endophilin-BAR and *Drosophila* amphiphysin-BAR strongly impedes liposome binding and tubulation [9], [11]. However, different from *I10E*, *EEE* has no effect on curvature sensing [23]. Hence, our finding that the *EEE* transgene failed to rescue the development of endoA nulls suggests that curvature sensing alone is not sufficient for endophilin-A to fulfill its function in synaptic endocytosis. An active function, likely to be curvature induction, is also required.

### Role of the Central Appendage/Hydrophobic Ridge

Our most surprising finding relates to the charge-negative substitution *A66D* and the hydrophilic substitutions *SS* and *SSQ*, which target the hydrophobic ridge in the central appendage. While these mutations all severely disrupt endophilin-A BAR tubulation activity in reduced systems, neither of them prevented development of the fly rescuants to adulthood, nor did they significantly impede sustained synaptic activity at the larval NMJ. It is clear from our study that the sensitivity to EndoA-BAR mutations is lower in larvae than in adults. However, even larvae are generally sensitive to *endoA* perturbation, as pure *endoA* nulls die early and with a severely disrupted NMJ function [5]. Hence, the complete lack of electrophysiological or locomotor effects in *A66D*, *SS* and *SSQ* larval rescuants remains surprising, in view of the strong in vitro effects reported for these mutations. The discrepancy between the in vitro and in vivo findings is most pronounced for the *A66D* mutation. Whereas *A66D* abolishes the tubulating capability of hEndoA1-BAR in the liposome assay [8], *A66D* in dEndoA-BAR had no significant effect in flies, not even in the adult rescuants.

Based on our results with *SS*, *SSQ* and *A66D*, it is tempting to conclude that in vitro tubulation does not always correlate well with BAR domain function in vivo, and indeed we suspect this to be the case. However, although we consider it unlikely in view of the high degree of structural conservation of the endophilin-A BAR domain, discrepancies between our in vivo findings and those predicted from work in reduced systems could relate to differences between the mammalian and fly endophilin proteins. Specifically, the tubulating activity of *Drosophila* EndoA-BAR wild type and mutant proteins has not been directly assayed. Therefore, the possibility remains that the tubulation activity of the fly protein is less sensitive to mutational perturbation than the activity of the mammalian orthologues, in accordance with our in vivo findings. In that case, the correlation between tubulation activity and in vivo function would still hold. Even so, it is impossible to escape the conclusion that at least at one synapse, the fly larval NMJ, complete structural integrity of the region homologous to the hydrophobic ridge of endophilin A is not of critical functional importance.

Despite the failure of the *A66D* mutation to exert any effect at all, and the missing effect of *SS* and *SSQ* in larvae, our results confirm that the central appendage/hydrophobic ridge *can* have an important role in vivo, as suggested by biochemical and cell culture studies [8], [9]. First, a clear negative effect was exerted by the *SS* and *SSQ* mutations in adults (contrasting the missing impact of these mutations in larvae). Second, more extensive mutations targeting the central appendage either completely disrupted (*ΔLQPNP*) or reduced (*EndoA(Arf)-HA*) the in vivo function of endophilin-A. We cannot exclude that the folding or dimerization of the proteins encoded by the unsuccessful transgenes was affected more than predicted from the alterations in primary structure. Specifically, a deletion of the central appendage of rat endophilin-A1 (residues 59–87), although it does not perturb the secondary structure, disrupts the dimerization of endophilin-A BAR [9]. The *ΔLQPNP* deletion was less extensive but may have had a similar effect. If loss of dimerization is associated with an elevated rate of degradation, this could explain the weak EndoA*^ΔLQPNP^* immunosignal (Figure 4). By contrast, severe misfolding or complete loss of dimerization can be ruled out for EndoA(Arf)-HA. First, this mutant was, after all, associated with a strong immunosignal, significant rescue of larval synaptic physiology and residual function in adult rescuant lines. Second, although more indirect, proper folding of the homologous mutant hEndoA1(Arf) was verified by X-ray crystallography [8].

### Role of the BAR Domain Curvature

No rescue was obtained with chimeras in which the linker region and SH3 domain of EndoA were combined with the N-BAR domain of amphiphysin or the F-BAR domain of either CIP4 or FCHo2. One explanation for this negative outcome might be the lack of a central appendage in these chimeras [24], [25; see the previous paragraph]. Still, a function at least similar to that in hEndoA1(Arf) rescuants might have been expected. A potential additional reason for the failure of the F-BAR chimeras is the weaker curvature of the F-BAR domain, compared to that of the N-BAR domain. This idea fits with the failure to rescue the *endoA nulls* that was also observed for the *endoA^P143A, ΔLEN^* transgene, a construct designed to decrease the curvature of the BAR domain without perturbing the central appendage. By contrast, a transgene mutated to *increase* the curvature (*endoA^D151P^*) rescued the endoA nulls as efficiently as the wild type transgene. One interpretation of these results is that a certain minimal degree of bending is demanded for proper function, but that the constraint imposed on the BAR curvature is otherwise relatively relaxed. During membrane re-modeling, proteins endowed with high-curvature (N-BAR) and low-curvature (F-BAR) domains segregate from each other on the membrane surface [27]. Hence, the loss-of-function of both EndoA*^P143A, ΔLEN^* and the EndoA/F-BAR chimeras may be linked to an inability of these “curvature-challenged” mutants to localize properly to the sites of presynaptic endocytosis.

An argument against the idea that weaker BAR domain curvature contributes to the rescuing failure of the F-BAR/EndoA chimeras is that the Amph-BAR/EndoA chimera was also unsuccessful. The curvature of the BAR scaffold is similar in amphiphysin and endophilin-A [9], [11]. However, other important aspects of the N-BAR domains of the two proteins might differ. For example, more negative charges are found on the convex surface of amphiphysin-BAR than on endophilin-BAR [9]. This could negatively affect the interaction with regulatory proteins “tuned” to interact with the endophilin-BAR. Also, Western blotting left the impression of a somewhat reduced expression/stability of EndoA*^Amph-BAR/EndoA-HA^* (Figure 4B).

### The A66W Mutation

The *A66W* mutation produced conspicuous and in some respects unique effects. First, expression of UAS-*endoA^A66W^* integrations, controlled by the *elav-GAL4* driver, was lethal in most cases. Notably, several of the UAS-*endoA^A66W^* integrations killed their hosts already at early embryonic stages. The lethality associated with UAS-*endoA^A66W^* was observed both in the absence of endogenous EndoA (in *endoA* nulls), and in the presence of EndoA in normal dosage (overexpression paradigm). Second, expression of *UAS-endoA^A66W^*, driven by *elav-GAL4*, was associated with a developmental eye defect (see below). The mechanism underlying these in vivo consequences of the *A66W* mutation is currently unknown. Interestingly, when hEndoA1-BAR*^A66W^* is added to liposomes in vitro, initial tubulation quickly gives way to massive vesiculation, indicating that *A66W* strongly enhances the curvature-inducing capability of endophilin-BAR [8]. This result raises the possibility that the *A66W* effect in vivo might be associated with an increased rather than decreased membrane internalization. More experiments are needed to substantiate this idea.

### A66W and Eye Development

How might the *A66W* mutation interfere with eye development? Several signaling pathways are implicated in the intimate link known to exist between clathrin-dependent endocytosis and *Drosophila* development [32]. A small-eye phenotype could be caused either by insufficient cell proliferation or exaggerated apoptosis in the eye disc. As regards the latter possibility, activation of the epidermal growth factor receptor (EGFR) recruits a complex that includes endophilin-A1 to the activated EGFR. This leads to clathrin-dependent endocytosis of the EGFR and subsequent proteolytic degradation [33], [34]. Inhibition of the EGFR/Ras1/MAPK pathway enhances cell killing activity and, like *endoA^A66W 4.1^*, causes an eye ablation phenotype [35]. Therefore, the eye phenotype that we observe might be explained by enhanced membrane internalization in the *EndoA^A66W^* mutant, leading to reduced activity of the EGFR-associated pathway and deregulation of apoptosis. Other signaling pathways related to endocytosis might also be involved, such as those including Notch [32], [36], [37] and the stress kinase c-Jun N-terminal kinase (JNK) [38], [39]. At this point it is also not possible to completely rule out that the *endoA^A66W^* product has an “unspecific” toxic effect that does not directly relate to the normal function of endophilin. However, the *endoA^A66W^* product must retain some endophilin-like function, since it rescued *endoA* nulls to pharate adults and also restored synaptic endocytosis at the NMJ.

In conclusion, we have provided novel information about the primary structure/function relationship of the endophilin-A BAR domain in vivo. Our results clearly validate the in vivo relevance of analyzing BAR domains under reduced conditions. However, extrapolation of conclusions drawn from such work to the behaving intact organism must be done with caution.
