# Correction: A Mutational Analysis of the Endophilin-A N-BAR Domain Performed in Living Flies

**DOI:** 10.1371/annotation/ce836c86-b5fa-43a4-be1c-3f0022db9a2d

**Published:** 2010-03-23

**Authors:** Anita G. Jung, Christina Labarrera, Anna M. Jansen, Klaus Qvortrup, Klemens Wild, Ole Kjaerulff

The second author's name is incorrect. The correct name is Christina Labarrera. The citation now reads: Jung AG, Labarrera C, Jansen AM, Qvortrup K, Wild K, et al. (2010) A Mutational Analysis of the Endophilin-A N-BAR Domain Performed in Living Flies. PLoS ONE 5(3): e9492. doi:10.1371/journal.pone.0009492

